# GPX8 regulates pan-apoptosis in gliomas to promote microglial migration and mediate immunotherapy responses

**DOI:** 10.3389/fimmu.2023.1260169

**Published:** 2023-09-19

**Authors:** Zigui Chen, Dandan Zheng, Ziren Lin, Chunyuan Zhang, Cheng Wei, Xiandong Deng, Peng Yan, Chuanhua Zheng, Chuanliu Lan, Chengjian Qin, Xuanlei Wei, Deling Qin, Yongfang Wu, Jun Peng, Changfeng Miao, Liuxue Lu, Ying Xia, Qisheng Luo

**Affiliations:** ^1^ Department of Neurosurgery, Affiliated Haikou Hospital of Xiangya Medical School, Central South University, Haikou, China; ^2^ Department of Radiation Oncology, The First Affiliated Hospital Zhejiang University, Hangzhou, China; ^3^ Department of Wound Repair Surgery, The People’s Hospital of Baise, Baise, Guangxi, China; ^4^ Life Science and Clinical Research Center, Affiliated Hospital of Youjiang Medical University for Nationalities, Baise, Guangxi, China; ^5^ Department of Neurosurgery, Affiliated Hospital of Youjiang Medical University for Nationalities, Baise, Guangxi, China; ^6^ Department of Laboratory Medicine, Neurosurgery Second Branche, The First Affiliated Hospital of Hunan Normal University, Changsha, Hunan, China; ^7^ Department of Medicine, Affiliated Hospital of Youjiang Medical University for Nationalities, Baise, Guangxi, China

**Keywords:** machine learning, non-apoptotic cell death, immunotherapy, glioma, pan-apoptosis

## Abstract

**Introduction:**

Gliomas have emerged as the predominant brain tumor type in recent decades, yet the exploration of non-apoptotic cell death regulated by the pan-optosome complex, known as pan-apoptosis, remains largely unexplored in this context. This study aims to illuminate the molecular properties of pan-apoptosis-related genes in glioma patients, classifying them and developing a signature using machine learning techniques.

**Methods:**

The prognostic significance, mutation features, immunological characteristics, and pharmaceutical prediction performance of this signature were comprehensively investigated. Furthermore, GPX8, a gene of interest, was extensively examined for its prognostic value, immunological characteristics, medication prediction performance, and immunotherapy prediction potential.

**Results:**

Experimental techniques such as CCK-8, Transwell, and EdU investigations revealed that GPX8 acts as a tumor accelerator in gliomas. At the single-cell RNA sequencing level, GPX8 appeared to facilitate cell contact between tumor cells and macrophages, potentially enhancing microglial migration.

**Conclusions:**

The incorporation of pan-apoptosis-related features shows promising potential for clinical applications in predicting tumor progression and advancing immunotherapeutic strategies. However, further in vitro and in vivo investigations are necessary to validate the tumorigenic and immunogenic processes associated with GPX8 in gliomas.

## Introduction

Gliomas account for the majority of malignant brain tumors, with approximately 81% falling into this category (1). Glioblastoma (GBM), the most aggressive histological subtype of glioma, exhibits a dismal 5-year survival rate, while low-grade gliomas (LGG) have a relatively better prognosis with around 50% survival rate (1). The significant morbidity and mortality associated with gliomas have spurred extensive research efforts dedicated to enhancing the diagnosis, treatment, and management of glioma patients (2). Genomic analyses, leveraging the power of big data, have provided accumulating evidence on the risk and prognosis of gliomas. Notably, the discovery of biomarkers such as MGMT, IDH, and 1p19q codeletion has improved survival outcomes for glioma patients. These remarkable findings have prompted numerous studies to explore new biomarkers based on different signature genes in gliomas (3–8).

Pyroptosis, apoptosis, and necroptosis have recently gained prominence as well-defined programmed cell death pathways that potentially influence glioma progression and response to cancer immunotherapy. These pathways actively participate in both physiological and pathological conditions, with pattern recognition receptors and inflammatory cytokine-induced signaling playing crucial roles in their activation (9). Furthermore, growing evidence has illuminated extensive crosstalk among these pathways, giving rise to the concept of pan-optosis (10). The pan-optosome complex, characterized by shared and distinct features of pyroptosis, apoptosis, and necroptosis, governs pan-optosis. Consequently, pan-optosis cannot be solely attributed to any single pathway (pyroptosis, apoptosis, or necroptosis) alone. Studies have implicated interferon regulatory factor 1, phosphorylated NFS1, and ADAR1 in the regulation of pan-optosis, demonstrating their roles in preventing colorectal cancer progression, influencing chemosensitivity, and promoting tumorigenesis, respectively (11–13). However, the specific roles of pan-optosis in gliomas remain inadequately addressed, highlighting the need for a comprehensive delineation of this phenomenon in the context of gliomas.

Evasion of immune surveillance poses a major challenge in glioma treatment, as these tumors create an immunosuppressive microenvironment that impairs immune cell function and hampers their ability to recognize and eliminate cancer cells. Recent studies have shed light on the impact of non-apoptotic cell death processes in gliomas on the immune response, potentially influencing the efficacy of immunotherapeutic approaches.

In this study, we aimed to depict the molecular features of pan-optosis-related genes in glioma patients. We grouped glioma patients based on the expression values of these genes and employed machine learning techniques to develop a pan-optosis-related signature. Systematic exploration was conducted to assess the prognostic value, mutation patterns, immune features, and drug prediction performance associated with this score. Notably, our findings identified GPX8 as a tumor promoter in gliomas.

## Method

### Data collection

The transcriptome data and clinical details of the glioma patients were accessed using two databases, The Cancer Genome Atlas (TCGA) and Chinese Glioma Genome Atlas (CGGA). The follow-up analysis used the TCGA and CGGA datasets.

Mutation analysis of pan-optosis-related genes:

To identify significantly altered single nucleotide polymorphisms (SNPs), the R package maftools were employed.

### Identification of pan-optosis-related clusters

The pan-optosis-related clusters were determined using the partition around medoids (PAM) approach from the R package clusterProfiler.

### Construction of pan-optosis-related signature

We identified the differentially expressed genes (DEGs) between two clusters associated with PANoptosis. For dimension reduction, the Random Survival Forest algorithm was applied. The prognostic DEGs were discovered using a single-variable Cox regression analysis. The PANoptosis-related score was also created using the dimension reduction and least absolute shrinkage and selection operator (LASSO) algorithms. The expression value of the gene × coefficient was used to construct the PANoptosis-related score.

### Mutation analysis of pan-optosis-related signature

Significantly amplified or deleted copy number variations (CNVs) were identified using GISTIC 2.0. The R package maftools was used to identify considerably altered SNPs.

### Pathway annotation

The correlation between the PANoptosis-related score and immune infiltrating cells (MCPcounter algorithm(14), ssGSEA algorithm(15), TIMER algorithm(16)) and immune modulators were analyzed. The gene set enrichment analysis (GSEA) of gene ontology (GO) and Kyoto Encyclopedia of Genes and Genomes (KEGG) terms was performed on the PANoptosis-related score. The gene set variation analysis (GSVA) of GO and KEGG terms was performed on GPX8. Estimation of STromal and Immune cells in MAlignant Tumor tissues using Expression data (ESTIMATE) algorithm was used for calculation of the microenvironment scores(17). T cell-inflamed gene expression profile (GEP), Cytotoxic activity (CYT), and Tumor mutation burden (TMB) were collected (18–20). Powerful software called Submap is used to foresee the outcome and responsiveness of immunotherapy against PD-1 and CTLA-4 related to GPX8 (21).

### Drug prediction

To predict candidate chemotherapy agents, the R package oncoPredict was utilized (22).

### Single-cell RNA sequencing (scRNA-seq) analysis on GPX8

SCP50 and SCP393’s scRNA-seq data were obtained via the Single Cell Portal website (http://singlecell.broadinstitute.org). The scRNA-seq matrix was processed using the R package Seurat. Malignant and non-malignant cells, respectively, were annotated using the R packages copykat and scCATCH. To ascertain the cell communication pattern, the R program iTalk was utilized.

### Cell culture

The human U251 and HMC3 cell lines were acquired from iCell (http://www.icellbioscience.com/search). U251 cells were cultured in DMEM medium supplemented with 10% fetal bovine serum (FBS) and 1% double antibody at 37°C with 5% CO2 in an incubator with saturated humidity. HMC3 cells were cultured in 1640 medium containing 10% FBS and 1% double antibody under the same incubation conditions.

### q-PCR assay

Primer design was conducted using Primer 5.0. The primer sequences and product lengths were as follows: β-actin (F: ACCCTGAAGTACCCCATCGAG, R: AGCACAGCCTGGATAGCAAC, product length: 224bp) and GPX8 (F: GCCCAGAGCAAAGGTTTCACTA, R: TTTGTGCAGTTCCTTCAGCCC, product length: 91bp). The siRNA sequences were used as follows: GPX8-Homo-142 GUCUAUAGUUCUAUGCACATT UGUGCAUAGAACUAUAGACTT

GPX8-Homo-324 GAAAUUACUUAGGGCUGAATT UUCAGCCCUAAGUAAUUUCTT

GPX8-Homo-664 GGUUAGACAAGUGAUCAUATT UAUGAUCACUUGUCUAACCTT.

### Cell counting kit-8 assay

In 96-well plates with a density of 2 × 10^3^ cells/well and 100 μL in each well, the cells were digested and counted. The corresponding treatment time was carried out using the aforementioned approach after the culture had been attached to the wall. The CCK8 solution was then set up with a full medium and 10 μL of CCK8 was added to each well. After a second two-hour incubation at 37°C with 5% CO2, the enzyme label from Bio-Tek measured the absorbance (OD) at 450 nm.

### EdU assay

The EdU solution (reagent A) was diluted 1000 times with cell culture media to create an adequate EdU medium of 50 μM. Cells were cultivated overnight in each well after adding 100 μL of 50 m EDU media. PBS was applied to the cells 1–2 times for 5 minutes. Each well received 50 μL of cell fixative (4% paraformaldehyde), which was then incubated for 30 minutes at room temperature. Each well received 50 mL of 2 mg/mL glycine, which was then incubated for 5 minutes in a shaker. Each well received PBS before being cleaned in the decolorization shaker for five minutes. Each decolorization well received 100 μL of penetrant, then incubated in a shaker for 10 min. Each well received 100 L of the 1 × Apollo^®^ staining solution, which was then incubated for 30 minutes at room temperature and away from light in a shaker. To decolorize the shaker and clean it twice for ten minutes each, 100 μL of penetrant was poured. Each well received 100 mL of methanol before being cleaned twice for five minutes each. To make 1 × Hoechst33342 reaction solution, reagent F was diluted 100:1 with deionized water. Each well received 100 μL of the 1× Hoechst 33342 reaction solution before being shaken for 30 minutes at room temperature and away from light. Each well received three additions of 100 μL PBS. The observation was done right away after the staining.

### Transwell assay

Matrigel was diluted to a final concentration of 200 μg per well using 100 μL of cold, serum-free DMEM media each well. A 500 μL 10% FBS complete medium was put to the lower container. Trypsin was used to break down the cells into single cells, which were then re-suspended at a concentration of 2 × 10^6^ cells per ml in a serum-free medium with 100 μL of cells added to each well. For 48 hours, the cells were incubated at 37°C. After being cleaned with PBS three times, the upper chamber was fixed for 20 minutes with 4% paraformaldehyde. The upper chamber was stained for 5 minutes with 0.1% crystal violet and then five times with water. An inverse microscope was used to examine the surface cells.

### Coculture for transwell assay

For the coculture Transwell assay, U251 cells were placed in the lower chamber with 500 μL of 10% FBS complete medium, while HMC3 cells were placed in the upper chamber with serum-free DMEM medium. The follow-up procedures were the same as mentioned above.

### Statistical analysis

Data analysis and visualization were performed using R software, version 4.1.2. We used the Wilcoxon rank sum test and the Kruskal-Wallis test, respectively, to analyze the differences between two groups and multiple groups for continuous variables. The Spearman correlation analysis was used to determine the correlation between two variables, and a two-sided hypothesis test was used to determine its significance. P-values below 0.05 were considered statistically significant (*p<0.05, **p<0.01, ***p<0.001, ****p<0.0001).

## Results

### Molecular features of pan-optosis-related genes

**Figure 1:**
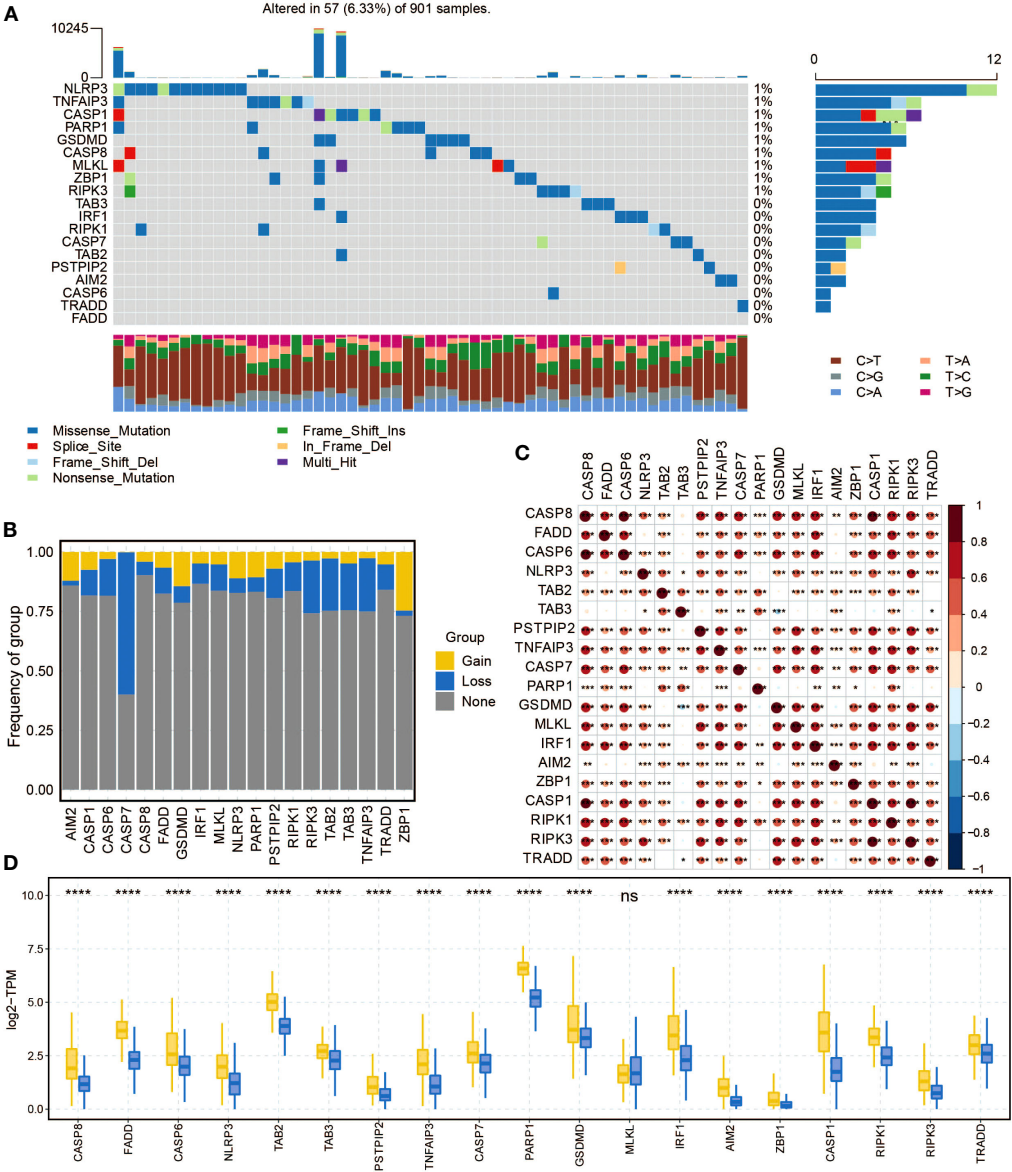
The molecular features of the pan-apoptosis-related genes. **(A)** Waterfall plot displaying the mutation frequency of the pan-apoptosis-related genes in the TCGA cohort. **(B)** Bar graph presenting the frequency of copy number variation (CNV) gain (yellow), CNV loss (blue), and CNV none (gray) of the pan-apoptosis-related genes in the TCGA cohort. **(C)** Heatmap illustrating the positive (red) and negative (blue) correlations among the pan-apoptosis-related genes in the TCGA cohort. **(D)** Box plot showing the expression differences of the pan-apoptosis-related genes in tumor samples and normal samples in the TCGA cohort. ns, not statistically significant; *P < 0.05; **P < 0.01; ***P < 0.001; ****P < 0.0001.

The mutation frequency of pan-optosis-related genes in the TCGA cohort was examined (**Figure 1A**). NLRP3, TNFAIP3, CASP1, PARP1, GSDMD, CASP8, MLKL, ZBP1, and RIPK3 showed a consistent mutation rate of 1%. Copy number variations (CNVs) of the pan-optosis-related genes were analyzed (**Figure 1B**), revealing different patterns of CNV gain, CNV loss, and CNV none. The correlation among pan-optosis-related genes was explored (**Figure 1C**), with most genes showing positive correlations. Additionally, the expression differences of pan-optosis-related genes between tumor and normal samples were investigated (**Figure 1D**), indicating higher expression levels in tumor samples.

### Construction of pan-optosis-related patterns

**Figure 2:**
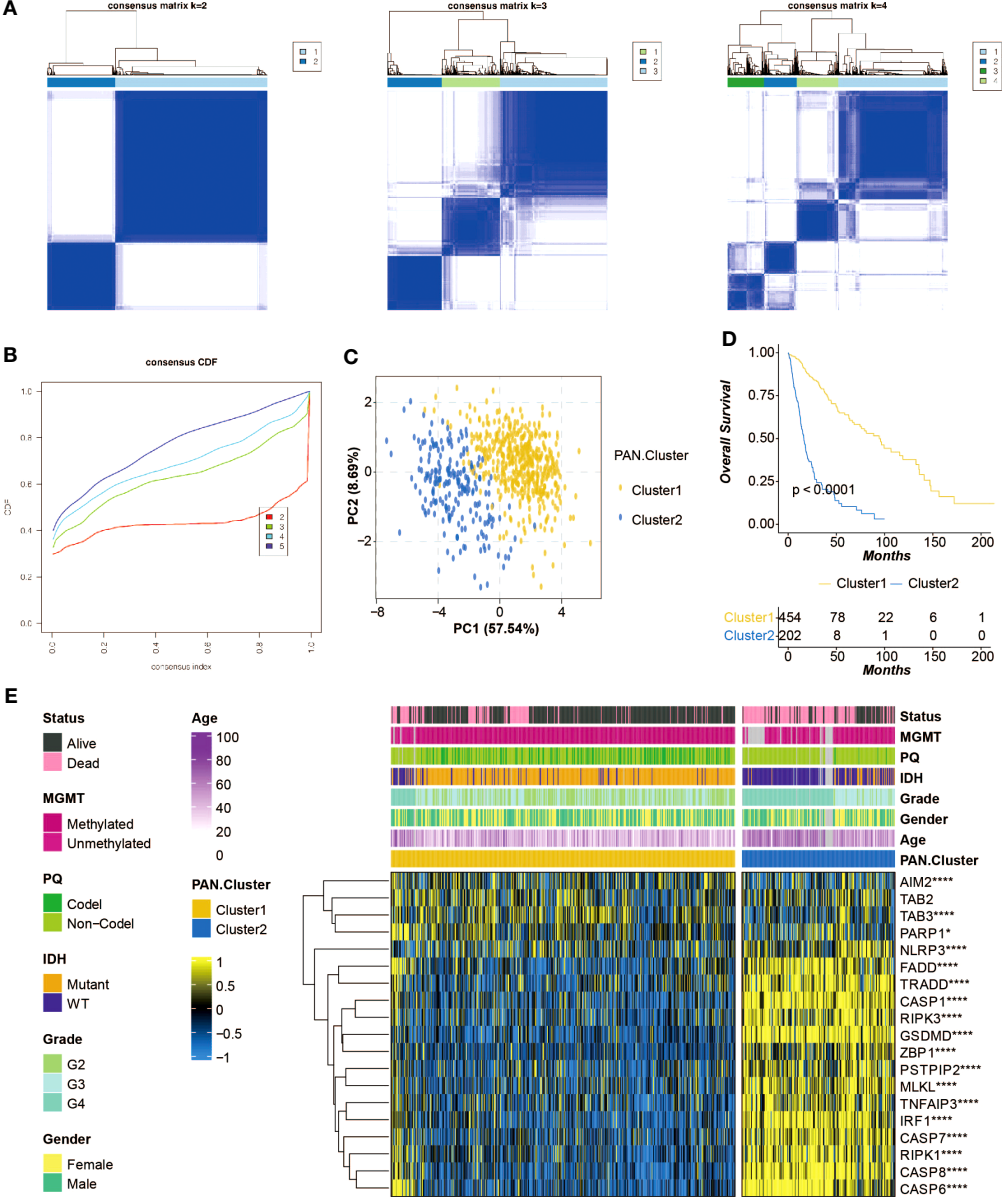
Construction of the pan-apoptosis-related clusters. **(A)** Consensus matrices of the pan-apoptosis-related clusters in the TCGA cohort. **(B)** Consensus cumulative distribution function (CDF) curves of the pan-apoptosis-related clusters in the TCGA cohort. **(C)** Principal Component Analysis (PCA) of the pan-apoptosis-related clusters in the TCGA cohort. **(D)** Survival curves of the pan-apoptosis-related clusters in the TCGA cohort. **(E)** Heatmap displaying the expression differences of the pan-apoptosis-related genes in the pan-apoptosis-related clusters in the TCGA cohort. ns, not statistically significant; *P < 0.05; ****P < 0.0001.

Consensus matrixes of the pan-optosis-related clusters were generated (**Figure 2A**), showing the clustering patterns of glioma patients. Consensus cumulative distribution function (CDF) curves were plotted to determine the optimal number of clusters (**Figure 2B**), resulting in the selection of k=2. Principal component analysis (PCA) demonstrated the separation of glioma patients within the pan-optosis-related clusters (**Figure 2C**). Survival curves of the pan-optosis-related clusters revealed distinct survival outcomes among the clusters (**Figure 2D**). The expression differences of pan-optosis-related genes within the clusters were analyzed (**Figure 2E**).

### Construction of pan-optosis-related signature

**Figure 3:**
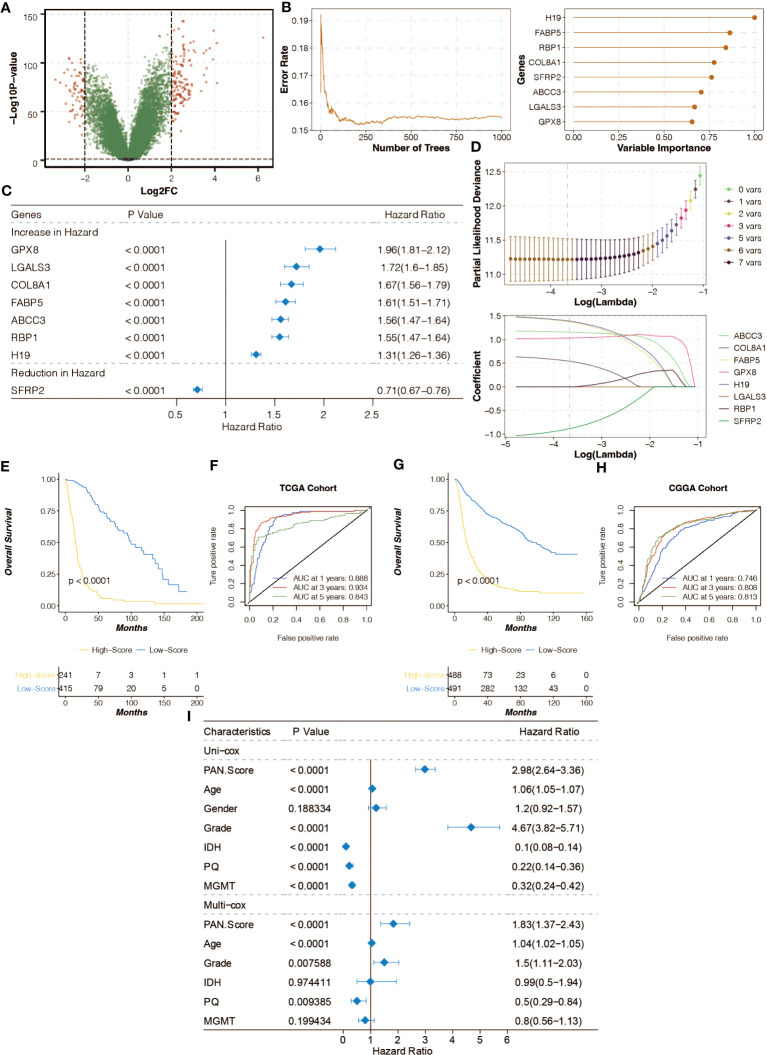
Construction of the pan-apoptosis-related signature. **(A)** Volcano plot showing the Differentially Expressed Genes (DEGs) between the pan-apoptosis-related clusters. **(B)** Random Survival Forest algorithm for dimension reduction of the DEGs in the TCGA cohort. **(C)** Univariate Cox regression analysis on the feature genes in the TCGA cohort. **(D)** Least Absolute Shrinkage and Selection Operator (LASSO) algorithm for dimension reduction of the feature genes and the construction of the pan-apoptosis-related signature in the TCGA cohort. **(E)** Survival curves of the pan-apoptosis-related signature groups in the TCGA cohort. **(F)** Receiver Operating Characteristic (ROC) curves of the pan-apoptosis-related signature groups in the TCGA cohort. **(G)** Survival curves of the pan-apoptosis-related signature groups in the CGGA cohort. **(H)** ROC curves of the pan-apoptosis-related signature groups in the CGGA cohort. **(I)** Univariate and multivariate Cox regression analysis on the clinical characteristics in the TCGA cohort.

Differentially expressed genes (DEGs) between the pan-optosis-related clusters were identified (**Figure 3A**). The Random Survival Forest algorithm was used for dimension reduction (**Figure 3B**), followed by univariate Cox regression analysis (**Figure 3C**). The LASSO algorithm was applied to construct the pan-optosis-related signature (**Figure 3D**). Survival curves of the pan-optosis-related signature groups indicated significant differences in survival outcomes (**Figure 3E**). Receiver operating characteristic (ROC) curves demonstrated the predictive performance of the pan-optosis-related signature (**Figure 3F**). The survival and ROC curves were also generated for the CGGA cohort (**Figures 3G, H**). Univariate and multivariate Cox regression analyses were performed on clinical characteristics (**Figure 3I**).

### SNP analysis of pan-optosis-related signature

**Figure 4:**
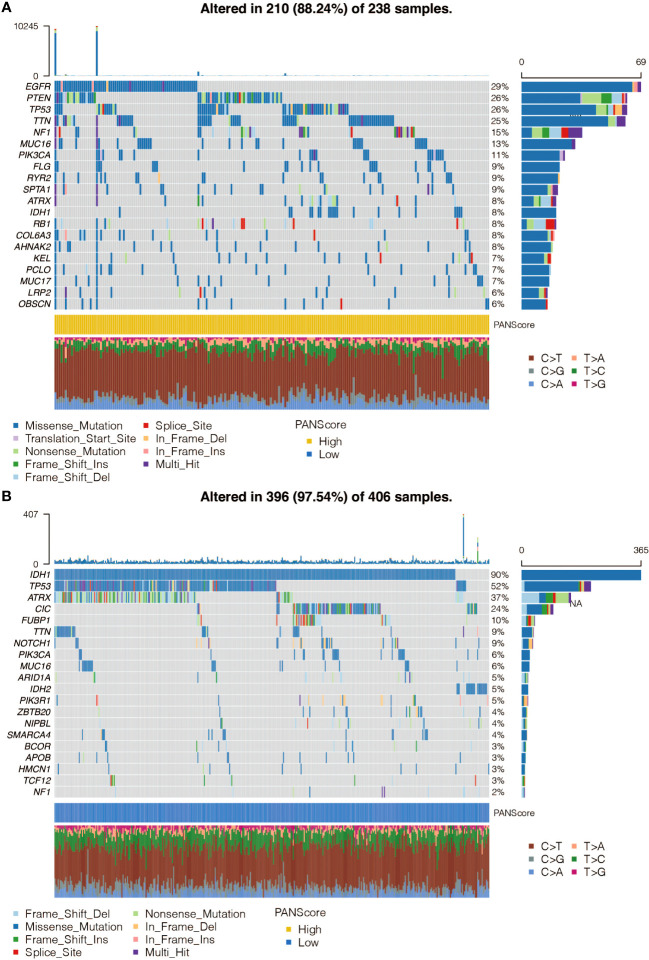
Single Nucleotide Polymorphism (SNP) analysis of the pan-apoptosis-related signature. **(A)** Waterfall plot showing the mutation frequency of the genes in the high pan-apoptosis-related signature group in the TCGA cohort. **(B)** Waterfall plot showing the mutation frequency of the genes in the low pan-apoptosis-related signature group in the TCGA cohort.

The mutation frequency of genes in the high and low pan-optosis-related signature groups was examined (**Figures 4A, B**). The differentially mutated genes and co-occurrence patterns between the two score groups were analyzed (**Figures S1A, B**).

### CNV analysis of pan-optosis-related signature

**Figure 5:**
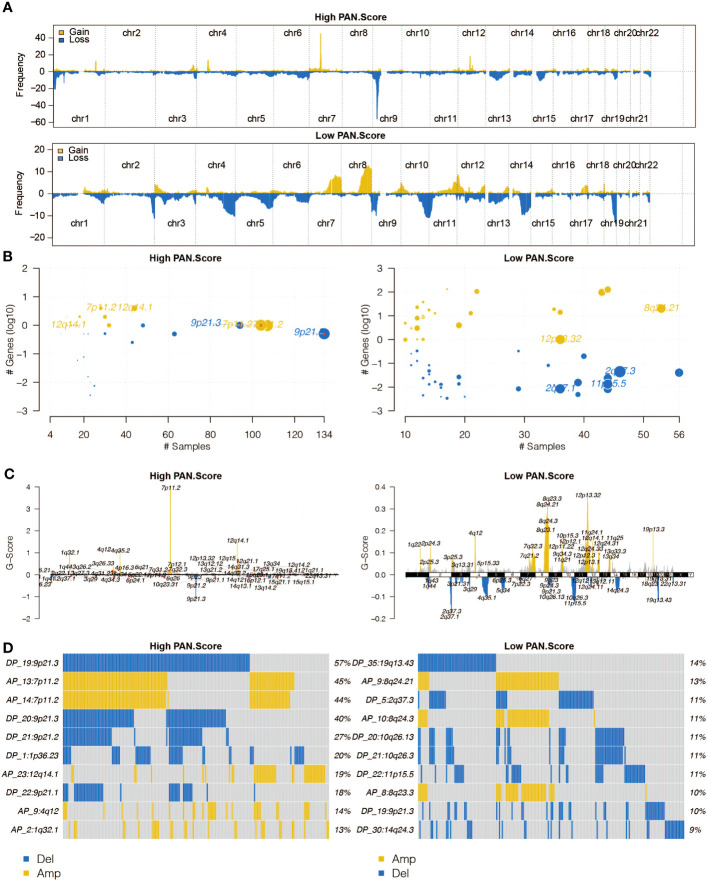
Copy Number Variation (CNV) analysis of the pan-apoptosis-related signature. **(A)** The overall altered chromosomes in the two pan-apoptosis-related signature groups in the TCGA cohort. **(B)** Bubble plot displaying the altered chromosomes in the two pan-apoptosis-related signature groups in the TCGA cohort. **(C)** Chromosome plot illustrating the altered chromosomes in the two pan-apoptosis-related signature groups in the TCGA cohort. **(D)** Oncoplot visualizing the altered chromosomes in the two pan-apoptosis-related signature groups in the TCGA cohort.

The altered chromosomes in the pan-optosis-related signature groups were investigated (**Figures 5A–D**), revealing significant differences between the groups.

### Immune analysis of pan-optosis-related signature

**Figure 6:**
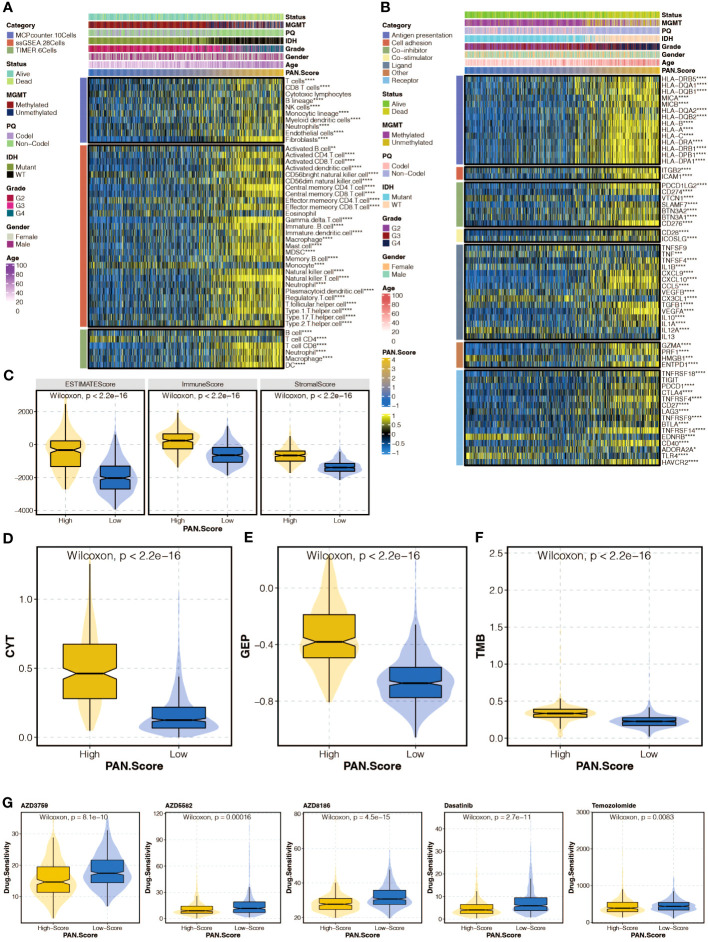
Immune analysis of the pan-apoptosis-related signature. **(A)** Heatmap depicting the correlation between the pan-apoptosis-related signature and immune infiltrating cells in the TCGA cohort. **(B)** Heatmap showing the correlation between the pan-apoptosis-related signature and immune modulators in the TCGA cohort. **(C)** Box plot presenting the levels of the microenvironment scores in the two pan-apoptosis-related signature groups in the TCGA cohort. **(D)** Box plot displaying the levels of Cytotoxic T lymphocytes (CYT) in the two pan-apoptosis-related signature groups in the TCGA cohort. **(E)** Box plot illustrating the levels of the Gene Expression Profile (GEP) in the two pan-apoptosis-related signature groups in the TCGA cohort. **(F)** Box plot demonstrating the levels of the Tumor Mutational Burden (TMB) in the two pan-apoptosis-related signature groups in the TCGA cohort. **(G)** Box plot showing the levels of the chemotherapy agents in the two pan-apoptosis-related signature groups in the TCGA cohort. ns, not statistically significant; *P < 0.05; **P < 0.01; ***P < 0.001; ****P < 0.0001.

The correlation between the pan-optosis-related signature and immune infiltrating cells was explored (**Figure 6A**). The association between the score and immune modulators was examined (**Figure 6B**). Microenvironment scores, including CYT, GEP, and TMB, were analyzed concerning the pan-optosis-related signature (**Figures 6C–F**). The drug sensitivity of the score groups to chemotherapy agents was evaluated (**Figure 6G**).

### Functional annotation of pan-optosis-related signature

**Figure 7:**
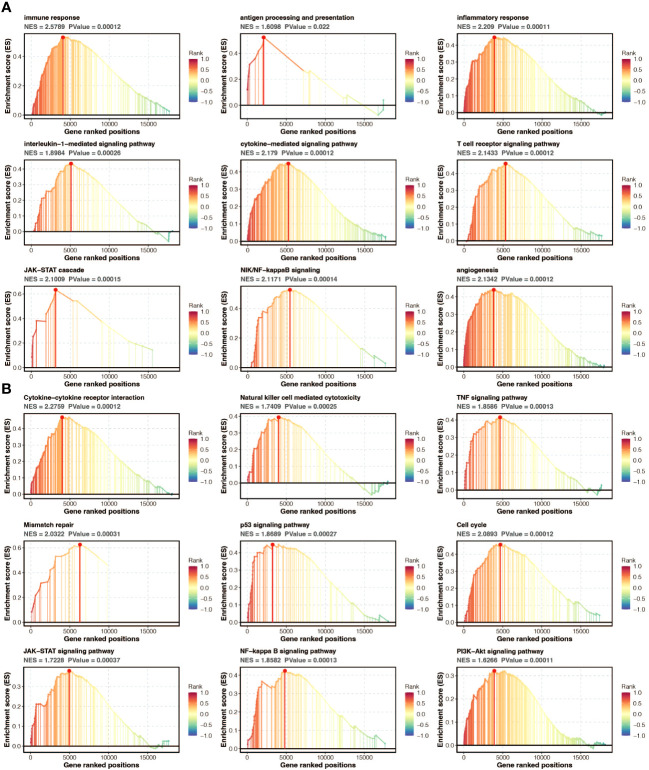
Functional annotation of the pan-apoptosis-related signature. **(A)** Gene Set Enrichment Analysis (GSEA) of Gene Ontology (GO) pathways on the pan-apoptosis-related signature. **(B)** GSEA of Kyoto Encyclopedia of Genes and Genomes (KEGG) pathways on the pan-apoptosis-related signature.

Gene set enrichment analysis (GSEA) of GO and KEGG pathways was performed to annotate the functional implications of the pan-optosis-related signature (**Figures 7A, B**).

### Molecular features of GPX8

**Figure 8:**
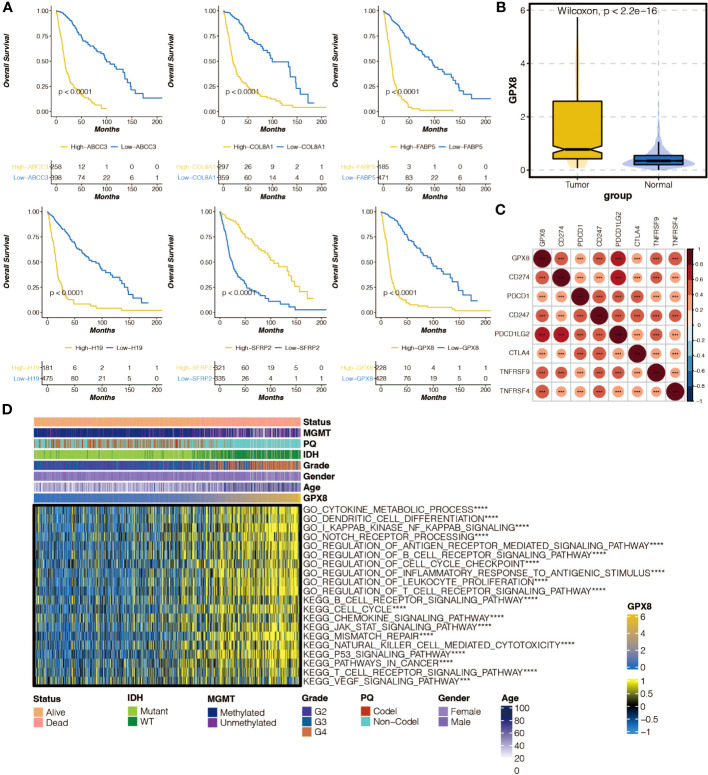
The molecular features of GPX8. **(A)** Survival curves of six model genes in the TCGA cohort. **(B)** Box plot showing the expression differences of GPX8 in tumor samples and normal samples in the TCGA cohort. **(C)** Heatmap illustrating the positive (red) and negative (blue) correlation between GPX8 and immune modulators in the TCGA cohort. **(D)** Heatmap displaying the positive (yellow) and negative (blue) correlation between GPX8 and Gene Set Variation Analysis (GSVA)-based GO and KEGG pathways in the TCGA cohort.ns, not statistically significant; ***P < 0.001; ****P < 0.0001.

Survival curves of model genes showed the impact of their expression on survival outcomes, including GPX8 (**Figure 8A**). The expression differences of GPX8 between tumor and normal samples were examined (**Figure 8B**). The correlation between GPX8 and immune modulators was analyzed (**Figure 8C**). The association between GPX8 and GSVA-based GO and KEGG pathways was explored (**Figure 8D**).

### Functional annotation of GPX8

GSEA of GO and KEGG pathways was performed to annotate the functional implications of GPX8 (**Figures S2A, B**).

### Immune analysis and immunotherapy prediction of GPX8

The correlation between GPX8 and immune infiltrating cells, as well as the levels of CYT, GEP, TMB, and drug sensitivity, were investigated (**Figures S3A–E**). The predictive value of GPX8 in immunotherapy response and its association with survival outcomes were analyzed across different cohorts (**Figures S4A–C**).

### *In vitro* validation on GPX8

**Figure 9:**
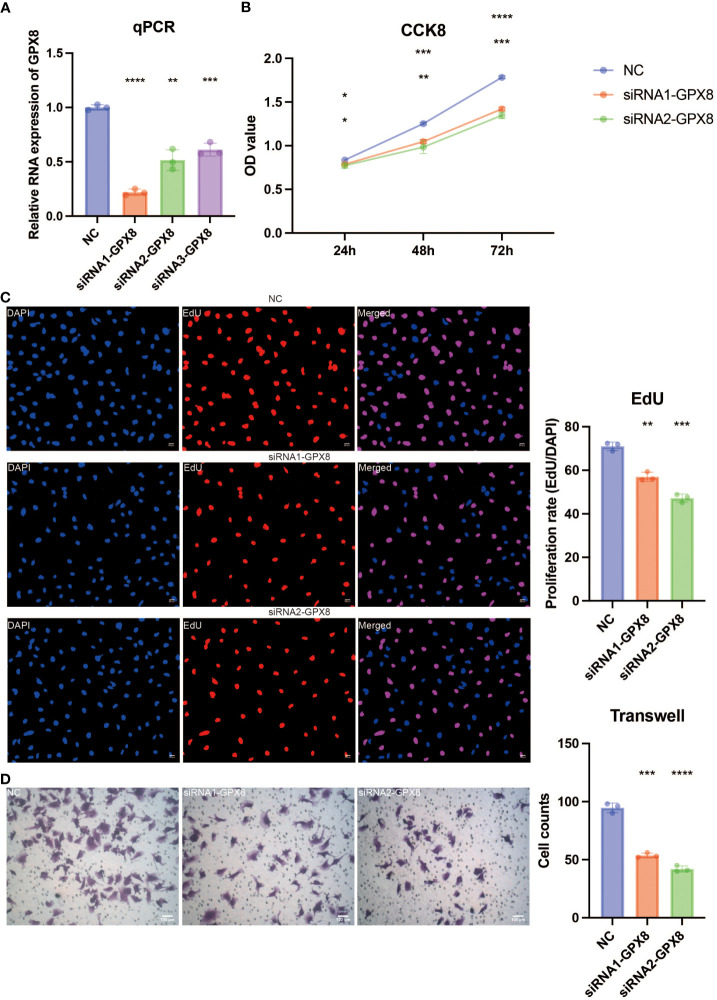
*In vitro* validation on GPX8. **(A)** Quantitative Polymerase Chain Reaction (qPCR) assay revealing the expression of GPX8 in four groups. **(B)** Cell Counting Kit-8 (CCK8) assay revealing the proliferation ability of U251 cells in three groups. **(C)** EdU assay revealing the proliferation ability of U251 cells in three groups. **(D)** Transwell assay revealing the migration ability of U251 cells in three groups. ns, not statistically significant; *P < 0.05; **P < 0.01; ***P < 0.001; ****P < 0.0001.

**Figure 10:**
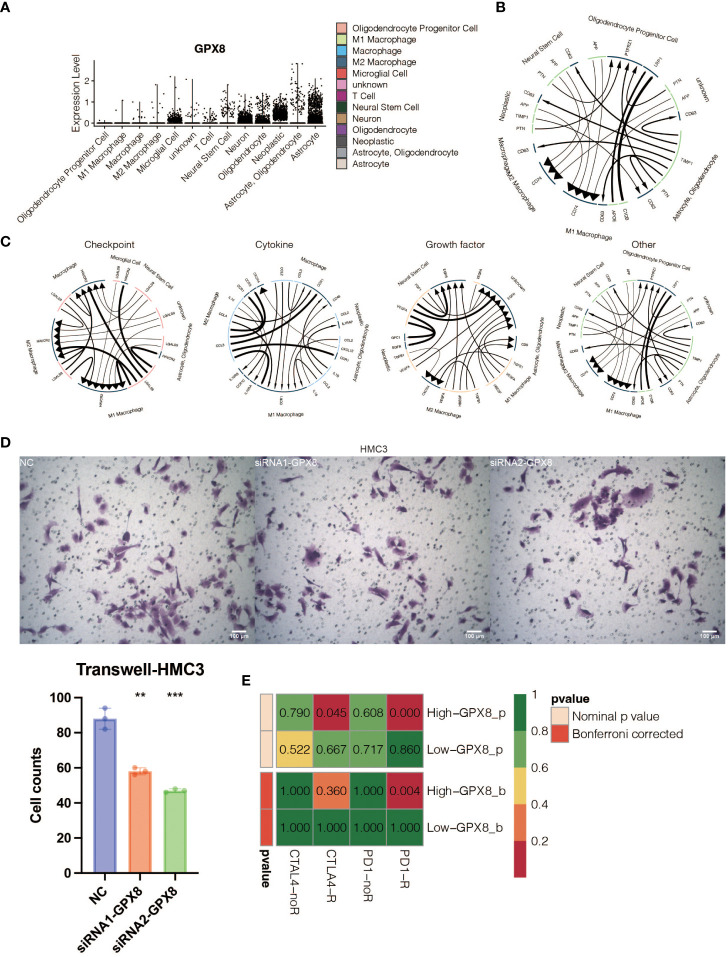
*In vitro* validation on GPX8. **(A)** Violin plot showing the expression pattern of GPX8 in 13 types of microenvironment cells identified at the single-cell RNA sequencing (scRNA-seq) level. **(B)** The general pattern of cell communication among GPX8-high microenvironment cells. **(C)** The manner in which cells in the microenvironment that express high levels of GPX8 communicate with one another regarding growth factors, cytokines, and checkpoints. **(D)** The Transwell assay demonstrated that HMC3 may migrate in three groups. **(E)** Submap analysis for anti-PD-1 and anti-CTLA-4 immunotherapy response prediction of GPX8. ns, not statistically significant; **P < 0.01; ***P < 0.001.

The pathogenic role of GPX8 in glioma was investigated in light of the exceptional performance of GPX8 in prognosis prediction. Three siRNA groups had considerably lower levels of GPX8 expression, according to the qPCR experiment (**Figure 9A**). For the follow-up tests, two siRNA with the best ability to inhibit the expression of GPX8 were employed. The CCK8 experiment showed that two siRNA groups greatly reduced the capacity of U251 cells to proliferate (**Figure 9B**). Two siRNA groups’ ability to proliferate U251 cells was severely reduced, according to the EdU assay (**Figure 9C**). According to the Transwell experiment, two siRNA groups dramatically reduced the capacity of U251 cells to migrate (**Figure 9D**). According to the scRNA-seq analysis, tumor cells and macrophages were shown to express GPX8 at high levels (**Figure 10A**). **Figure 10B** depicts the overall cell communication network involving macrophages and tumor cells in the microenvironment that express high levels of GPX8. **Figure 10C** illustrates the cell communication pattern between macrophages and tumor cells in the microenvironment that express high levels of GPX8 in terms of checkpoint, cytokine, growth factor, and other factors. Additionally, the Transwell assay using cocultured U251 and HMC3 cells showed that two siRNA groups’ ability to migrate HMC3 cells was dramatically reduced (**Figure 10D**). Besides, the submap analysis confirmed that high GPX8 expression was associated with better anti-PD-1 immunotherapy response (**Figure 10E**).

## Discussion

Pan-apoptosis is a unique innate immune inflammatory cell death modality (23). In the context of cancer, pan-apoptosis refers to the induction of apoptosis in cancer cells. This can be achieved through various mechanisms, such as activation of pro-apoptotic proteins or inhibition of anti-apoptotic proteins. By promoting apoptosis in cancer cells, pan-apoptosis can help to suppress tumor growth and prevent the spread of cancer. Additionally, pan-apoptosis can also enhance the effectiveness of cancer treatments, such as chemotherapy or radiation therapy, by sensitizing cancer cells to these therapies. Pan-apoptosis has been extensively studied as a biomarker for survival and immunotherapy response in gastric cancer (24). However, its specific roles in gliomas remain to be deciphered. Therefore, it is essential to systematically illustrate the prognostic and predictive values of pan-apoptosis-related genes in glioma patients by developing a pan-apoptosis-related signature.

To explore the molecular features of pan-apoptosis-related genes, a comprehensive analysis was performed. NLRP3, TNFAIP3, CASP1, PARP1, GSDMD, CASP8, MLKL, ZBP1, and RIPK3 were found to have a low mutation rate (1%) in gliomas. Among them, CASP7 showed the highest frequency of CNV gain and CNV loss. Furthermore, all pan-apoptosis-related genes exhibited higher expression levels in tumor samples. Subsequently, pan-apoptosis-related clusters were constructed based on these genes, and it was observed that the survival outcomes of glioma patients could be significantly separated by these clusters.

Using machine learning algorithms, an eight-gene pan-apoptosis-related signature was developed with robust prognostic value. The signature proved to be an independent prognostic marker, even after accounting for age, gender, grade, IDH, 1p19q, and MGMT status. In the high pan-apoptosis-related signature group, frequent mutations were detected in oncogenes such as EGFR, PTEN, and TTN. EGFR has been previously implicated in therapeutic interventions for GBM (25), while PTEN is a key mechanism and actionable target associated with radiation resistance in gliomas (26). On the other hand, tumor suppressors including IDH, TP53, and ATRX exhibited high mutation frequency in the low pan-apoptosis-related signature group. These mutations have been reported to play important roles in glioma progression and patient outcome (27, 28), supporting the hazardous nature of the pan-apoptosis-related signature.

The tumor microenvironment (TME) of gliomas plays a critical role in tumor progression and recurrence (29). The high pan-apoptosis-related signature group displayed higher microenvironment scores, including ESTIMATE score, Immune score, and Stromal score. Despite the notion that gliomas are generally considered cold tumors with a relatively inactivated immune response due to the unique characteristics of the central nervous system, increasing evidence suggests that the immune system does indeed function in the TME of gliomas due to disruption of the blood-brain barrier. Importantly, the pan-apoptosis-related signature showed positive associations with several immune infiltrating cells, including macrophages, mast cells, MDSCs, and fibroblasts. Moreover, immune response-related pathways and processes, such as antigen processing and presentation, inflammatory response, interleukin-1-mediated signaling pathway, cytokine-mediated signaling pathway, T cell receptor signaling pathway, JAK-STAT cascade, NIF/NF-kappaB signaling, and angiogenesis, were highly enriched in the high pan-apoptosis-related signature group. The immune escape of tumor cells, represented by the immune checkpoint, has been identified as a contributing factor to the limited efficacy of glioma treatments (30). However, immune checkpoint inhibitors have shown promise in promoting anti-glioma immune responses (30). Interestingly, the pan-apoptosis-related signature exhibited positive associations with several immune modulators, including CD274, CD276, VTCN1, PDCD1, and CTLA4. Furthermore, immunotherapy determinants, including CYT, GEP, and TMB, were higher in the high pan-apoptosis-related signature group. These findings collectively indicate the presence of an immune suppressive microenvironment in the high pan-apoptosis-related signature group.

Besides, the high PANoptosis-related score group had lower drug sensitivity towards AZD3759, AZD5582, AZD8186, Dasatinib, and Temozolomide. AZD3759 is a small molecule inhibitor of the epidermal growth factor receptor (EGFR) tyrosine kinase. It is being investigated for the treatment of non-small cell lung cancer (NSCLC) with EGFR mutations. AZD5582 is a small molecule inhibitor of the B-cell lymphoma 2 (BCL-2) protein. It is being studied for the treatment of various types of cancer, including lymphoma and leukemia. AZD8186 is a small molecule inhibitor of the phosphoinositide 3-kinase (PI3K) enzyme. It is being developed for the treatment of solid tumors with PI3K pathway alterations. Dasatinib is a tyrosine kinase inhibitor that targets multiple kinases, including BCR-ABL, SRC family kinases, and c-KIT. It is approved for the treatment of chronic myeloid leukemia (CML) and Philadelphia chromosome-positive acute lymphoblastic leukemia (Ph+ ALL). Temozolomide is an alkylating agent that is used for the treatment of glioblastoma multiforme, a type of brain cancer. It is also used in combination with other drugs for the treatment of certain types of melanoma.

Among the six genes included in the pan-apoptosis-related signature, LGALS3 has been reported as a novel biomarker for disease diagnosis and a therapeutic target (31). COL8A1 has been shown to promote non-small cell lung cancer progression by activating IFIT1/IFIT3-mediated EGFR signaling (32), while FABP5 can promote lymph node metastasis in cervical cancer by reprogramming fatty acid metabolism (33). ABCC3 has been proposed as a potential therapeutic target in cancer (34), and the RBP1-CKAP4 axis has been found to activate oncogenic autophagy and promote cancer progression in oral squamous cell carcinoma (35). H19 plays a critical role in regulating tumor plasticity in neuroendocrine prostate cancer (36), while SFRP2 in the aged microenvironment drives melanoma metastasis and therapy resistance (37).

GPX8, also known as glutathione peroxidase 8, is an enzyme that plays a role in protecting cells from oxidative stress. Oral cancer cells’ m6A epitranscriptome was altered by oxidative stress brought on by GPX8 deficiency(38). By stimulating the Wnt signaling pathway, GPX8 is transcriptionally controlled by FOXC1 and aids in the proliferation of gastric cancer cells(39). Through encouraging lipogenesis by NNMT, GPX8 controls the development of clear cell renal cell carcinoma tumors(40). However, when it comes to its involvement in cancer, particularly glioblastoma, there is limited information available. GPX8 has not been extensively studied in the context of glioblastoma, and its specific role in this type of cancer is not well understood. It’s important to note that glioblastoma is a complex and multifactorial disease, and the exact mechanisms underlying its development and progression are still being actively researched. The GPX8/IL-6/STAT3 axis has been shown to maintain an aggressive breast cancer phenotype (41), and GPX8 itself mediates the proliferation and migration of GBM cells. Furthermore, our study proved that GBM-derived GPX8 promotes the migration of HMC3 cells in a co-culture system.

The development of a transcriptomic biomarker based on pan-apoptosis, encompassing various non-apoptotic forms of cell death in gliomas, holds significant implications for predicting immunotherapeutic response. Analyzing the gene expression patterns associated with these non-apoptotic cell death processes may lead to the identification of specific molecular signatures that correlate with an immunologically active tumor microenvironment and a favorable response to immunotherapy. Such a biomarker has the potential to aid clinicians in stratifying patients and selecting those who are most likely to benefit from immunotherapy. Furthermore, it could guide the development of novel combination therapies targeting both apoptotic and non-apoptotic cell death pathways, thereby enhancing the efficacy of immunotherapy in gliomas. However, it is important to acknowledge that the development of transcriptomic biomarkers is a complex and iterative process, necessitating extensive validation in large patient cohorts. Additionally, the field of glioma immunotherapy is rapidly evolving, and new insights may emerge that further refine our understanding of the relationship between non-apoptotic cell death and immunotherapeutic response.

In conclusion, our study elucidates the crucial role of pan-apoptosis in glioma patients. By developing a pan-apoptosis-related signature, we have provided insights into the regulatory mechanisms underlying pan-apoptosis in gliomas. Moreover, this signature exhibits potential value in clinical applications. The tumorigenic and immunogenic mechanisms associated with GPX8 warrant further validation through *in vitro* and *in vivo* experiments in glioma models.

## Data availability statement

The original contributions presented in the study are included in the article/**Supplementary Material**, further inquiries can be directed to the corresponding author/s.

## Ethics statement

Ethical approval was not required for the studies on humans in accordance with the local legislation and institutional requirements because only commercially available established cell lines were used.

## Author contributions

ZC: Data curation, Funding acquisition, Writing – original draft, Writing – review & editing. DZ: Writing – review & editing. ZL: Writing – review & editing. CZhang: Writing – review & editing. CW: Writing – original draft. XD: Writing – original draft. PY: Writing – original draft. CZheng: Writing – original draft. CL: Writing – original draft. CQ: Writing – original draft. XW: Writing – original draft. DQ: Writing – original draft. YW: Writing – original draft. JP: Writing – original draft. CM: Supervision, Writing – review & editing. LL: Supervision, Writing – review & editing. YX: Funding acquisition, Supervision, Writing – review & editing. QL: Data curation, Funding acquisition, Supervision, Writing – review & editing.
