# Supplementary material for: GPX8 regulates pan-apoptosis in gliomas to promote microglial migration and mediate immunotherapy responses
Source: Front Immunol. 2023 Sep 19;14:1260169. doi: 10.3389/fimmu.2023.1260169 (PMC10545954; doi:10.3389/fimmu.2023.1260169)
Supplement: Supplementary file 1 [file DataSheet_1.docx]

Materials and Methods

Cell Counting Kit-8 (CCK8) assay:

CCK-8 assay, also known as the Cell Counting Kit-8 assay, is a widely used method in cell biology and drug discovery. It is used to measure cell viability and proliferation. The assay is based on the reduction of a yellow water-soluble tetrazolium salt, WST-8, to formazan by cellular dehydrogenases. The formazan produced is directly proportional to the number of living cells in the culture. This assay is commonly used to assess the cytotoxicity of drugs, evaluate cell proliferation, and determine cell viability. It is a quick and reliable method that provides valuable information about cell health and growth.

EdU assay:

EdU assay, also known as the 5-ethynyl-2'-deoxyuridine assay, is a widely used method to detect and quantify DNA synthesis in cells. It is based on the incorporation of EdU, a thymidine analog, into newly synthesized DNA during the S phase of the cell cycle. EdU is a small molecule that can be easily detected using a fluorescent azide through a copper-catalyzed click reaction. This assay offers several advantages over traditional methods, such as BrdU incorporation, including faster and more efficient detection, compatibility with various sample types, and minimal DNA denaturation. It has become a valuable tool in cell biology research for studying cell proliferation, DNA replication, and cell cycle dynamics.

Transwell assay:

Transwell assay is a commonly used in vitro technique that allows researchers to study cell migration and invasion. It consists of a porous membrane insert placed in a well, creating two separate compartments. Cells are seeded on the upper side of the membrane and a chemoattractant is added to the lower compartment. Over time, cells migrate through the pores of the membrane towards the chemoattractant. This assay is widely used in various fields, including cancer research, drug discovery, and developmental biology, to investigate cell behavior and evaluate the effects of different factors on cell migration and invasion.

Supplementary Figure


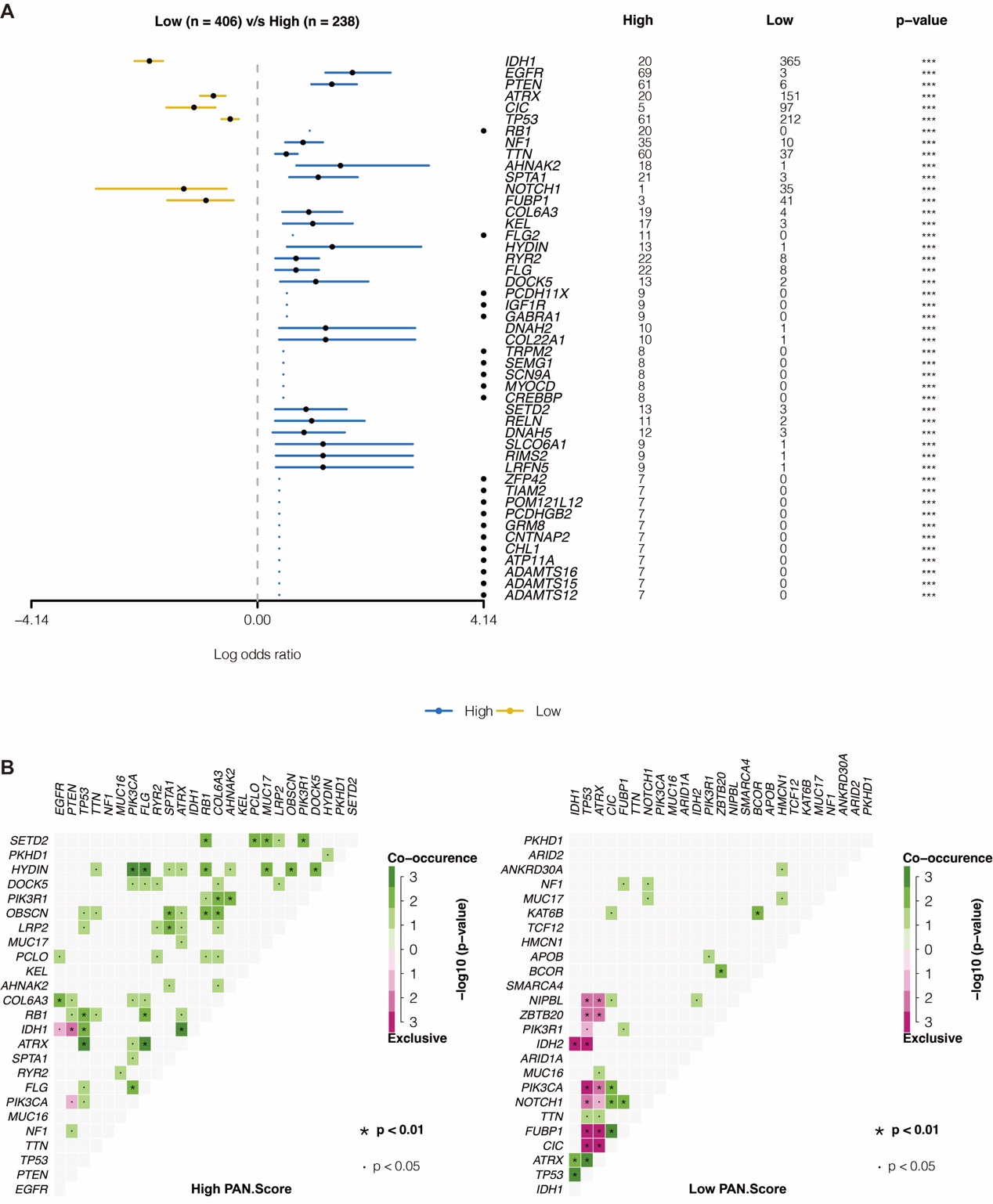


Figure S1. SNP analysis of the PANoptosis-related score. A. The differentially mutated genes in the two PANoptosis-related score groups. B. The mutually occurred and exclusive mutations in the two PANoptosis-related score groups.


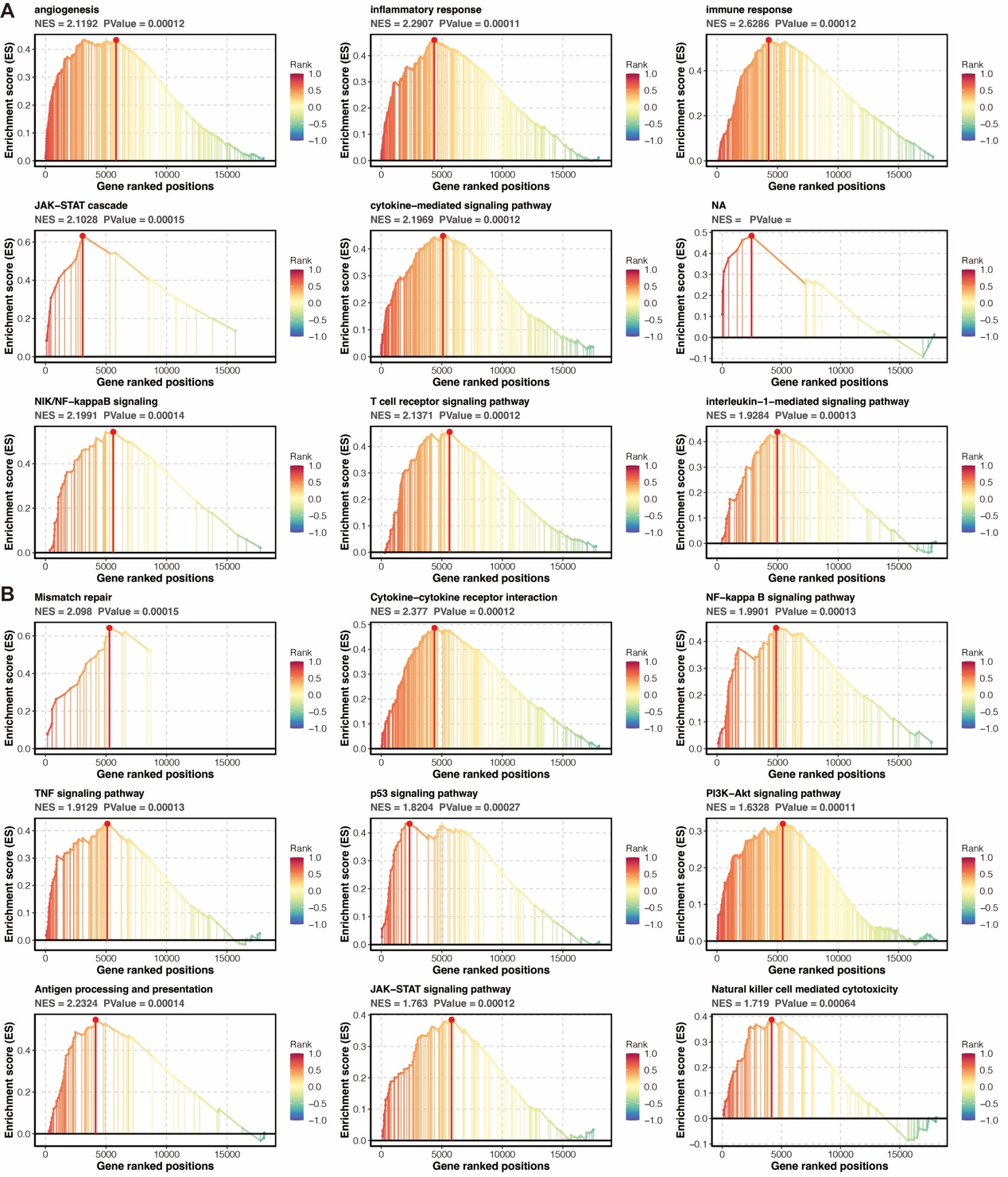


Figure S2. Functional annotation of GPX8.

A. GSEA of GO pathways on GPX8.

B. GSEA of KEGG pathways on GPX8.


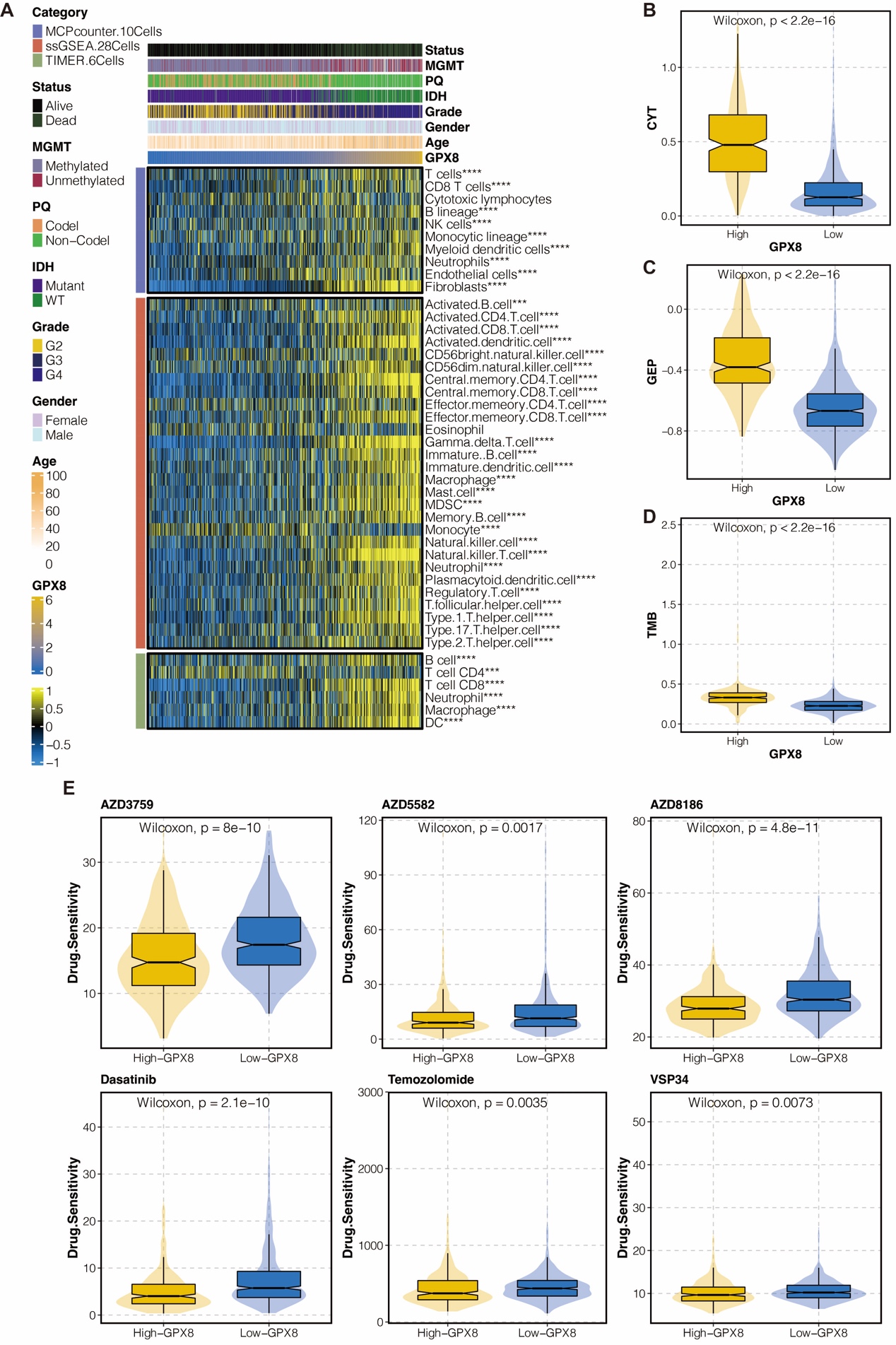


Figure S3. Immune analysis of GPX8.

A. Heatmap depicting the correlation between GPX8 and immune infiltrating cells in the TCGA cohort.

B. Box plot showing the levels of CYT in the two GPX8-related groups in the TCGA cohort.

C. Box plot displaying the levels of GEP in the two GPX8-related groups in the TCGA cohort.

D. Box plot illustrating the levels of TMB in the two GPX8-related groups in the TCGA cohort.

E. Box plot presenting the levels of the chemotherapy agents in the two GPX8-related groups in the TCGA cohort.


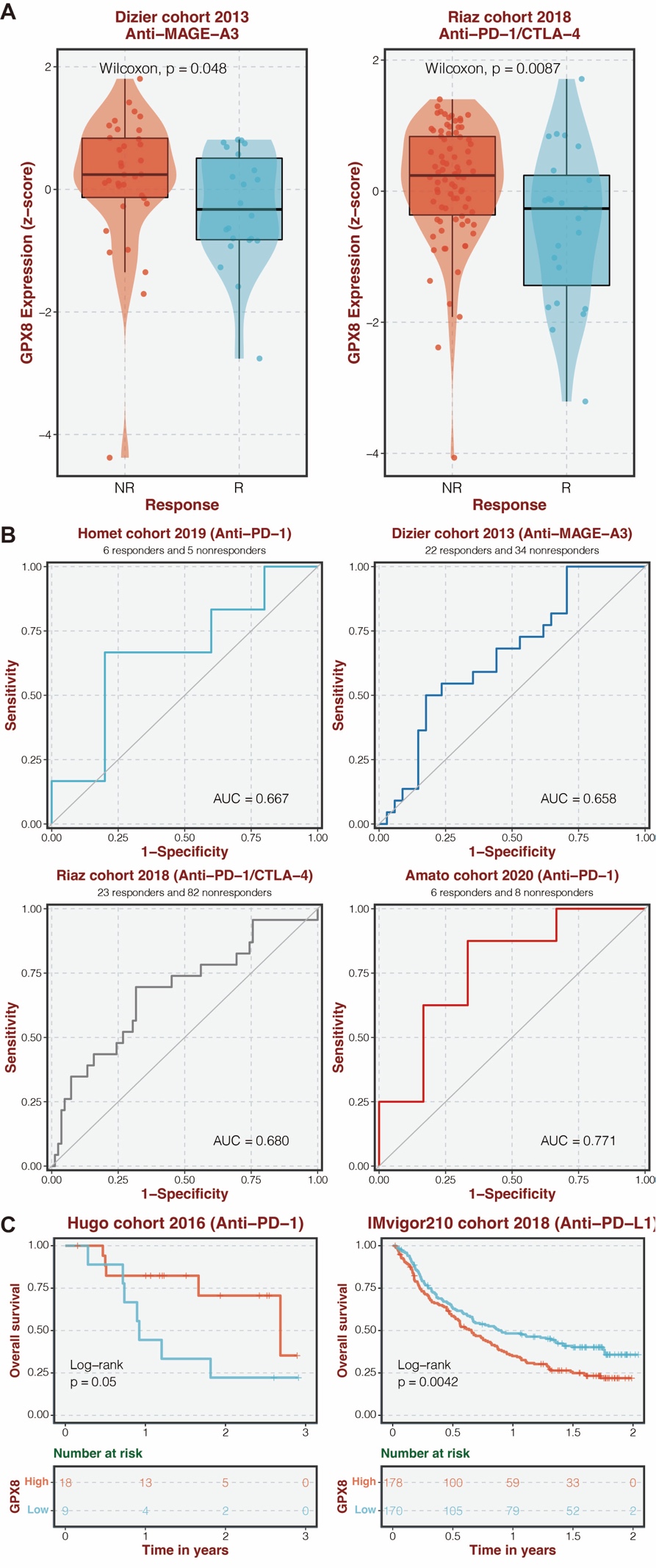


Figure S4. Immunotherapy prediction of GPX8.

A. Box plot showing the expression differences of GPX8 in responders and non-responders in two immunotherapy cohorts.

B. ROC curve regarding the sensitivity of GPX8 in immunotherapy prediction in four immunotherapy cohorts.

C. Survival curves of the two GPX8-related groups in two immunotherapy cohorts.
